# The relationshİp of socioeconomic status with sexual satisfaction through gender roles and sexual myths

**DOI:** 10.1192/j.eurpsy.2023.674

**Published:** 2023-07-19

**Authors:** T. Kizilakca Soyaslan, I. Ozgur Ilhan

**Affiliations:** ^1^Community Health Center, Kirikkale; ^2^Psychiatry, Ankara University Medical School, Ankara, Türkiye

## Abstract

**Introduction:**

Sexual life and sexual satisfaction are associated with psychological well-being. It has already been shown that sexual satisfaction is related to sexual myths or stereotypes, somesociodemographic and sociosultural variables, and gender. However, we have not met any study in which socioeconomic status, sexual myths and gender roles were taken together.

**Objectives:**

The aim of this study was to investigate whether socioeconomic status predicts sexual satisfaction through sexual myths and gender roles.

**Methods:**

The Bem Gender Roles Scale (Ozkan and Lajunen Sex Roles 2005;103-110), the Sexual Myths Scale (Golbasi et al. Sex Disabil 2016; 34 75-87), the New Sexual Satisfaction Scale (Stulhofera et al. J Sex Res 2010;47 257-268), the Socio-Economic Status Measurement Tool (Kalaycioglu et al. J Soc Res 2010; 1 183-220) were applied face-to-face or online to 185 women and 74 men who had heterosexual relationships. Relationships between scale scores were examined with Pearson correlation analysis. Serial multiple mediator analysis was used to test mediator role of either masculinity or sexual myths in the relationship between socioeconomic status and sexual satisfaction.

**Results:**

We found significant correlations between socioeconomic status and sexual myths (r=-.22, p<.001), between socioeconomic status and sexual satisfaction (r=.13, p<.001), sexual myths and sexual satisfaction (r=.20, p>.001) and between masculinity and sexual satisfaction (r=.18, p=.004), The relationship between femininity and sexual satisfaction was not significant (r=.01, p=.845). Sexual myths (b=-.19, t(257)=-3.48, p<.01) and masculinity (b=.40, t(257)=3.26, p<.01) mediated the relationship between socioeconomic status and sexual satisfaction (b=.15, t(257)= 2.04, p<.05).

**Conclusions:**

Interventions on sexual myths will reduce the effect of socioeconomic disadvantage on sexual satisfaction.

**Disclosure of Interest:**

None Declared

